# Predicting false lumen thrombosis in patient-specific models of aortic dissection

**DOI:** 10.1098/rsif.2016.0759

**Published:** 2016-11

**Authors:** Claudia Menichini, Zhuo Cheng, Richard G. J. Gibbs, Xiao Yun Xu

**Affiliations:** 1 Department of Chemical Engineering, Imperial College London, London SW7 2AZ, UK; 2 Department of Surgery and Cancer, St Marys Hospital, Imperial College Healthcare NHS Trust, London W2 1NY, UK

**Keywords:** aortic dissection, thrombus formation and growth, blood flow, computational model

## Abstract

Aortic dissection causes splitting of the aortic wall layers, allowing blood to enter a ‘false lumen’ (FL). For type B dissection, a significant predictor of patient outcomes is patency or thrombosis of the FL. Yet, no methods are currently available to assess the chances of FL thrombosis. In this study, we present a new computational model that is capable of predicting thrombus formation, growth and its effects on blood flow under physiological conditions. Predictions of thrombus formation and growth are based on fluid shear rate, residence time and platelet distribution, which are evaluated through convection–diffusion–reaction transport equations. The model is applied to a patient-specific type B dissection for which multiple follow-up scans are available. The predicted thrombus formation and growth patterns are in good qualitative agreement with clinical data, demonstrating the potential applicability of the model in predicting FL thrombosis for individual patients. Our results show that the extent and location of thrombosis are strongly influenced by aortic dissection geometry that may change over time. The high computational efficiency of our model makes it feasible for clinical applications. By predicting which aortic dissection patient is more likely to develop FL thrombosis, the model has great potential to be used as part of a clinical decision-making tool to assess the need for early endovascular intervention for individual dissection patients.

## 1. Introduction

Aortic dissection is a major aortic catastrophe with high morbidity and mortality that compromises blood perfusion in the entire body. Dissection occurs when a tear is formed on the inner layer of the aortic wall, allowing flow diversion through the tear within the aortic wall. The thrust of diverted blood causes separation of the wall layers, leading to the formation of a ‘false lumen’ (FL). Distal (Stanford type B) dissections are usually treated medically or through endovascular stenting. For these patients, a large variability exists in post-discharge prognosis with 5 year mortality rates varying between 48% and 82% [1]. Significant predictors for late outcomes include age and the status of the FL (patent, partially or completely thrombosed) [1,2]. Partial thrombosis of the FL has been associated with enlargement of the aortic diameter and post-discharge mortality, possibly owing to the occlusion of distal re-entry tears and increase in diastolic pressure [3], leading to high wall tension, which is a risk for expansion and rupture. On the other hand, multiple studies highlight the beneficial prognostic value of complete FL thrombosis.

The development of FL thrombosis in medically and endovascularly treated patients is largely affected by the distribution of haemodynamic parameters. Computational studies of aortic dissection have shown highly disturbed flow patterns, with large variability in wall shear stress, extended areas of flow recirculation and potential regions of stasis [4–6]. All these factors play a major role in the formation of thrombus [7–9]. The use of numerical simulations holds much promise in understanding what conditions favour FL thrombosis, and may help identify key parameters for prediction of thrombus growth and therapeutic outcomes, and for estimating the effectiveness of endovascular treatments. A large number of comprehensive models have been developed which include detailed descriptions of the chain of biochemical reactions and transport processes leading to the formation of thrombus [10–14]. However, these models are computationally very demanding, and their applications are often limited to steady flow, Newtonian fluid and two-dimensional fluid domains, making them unsuitable for patient-specific simulations. Other studies have focused on understanding the role of haemodynamic parameters and in particular shear stress on the initial formation of thrombus [14–16], although often neglecting its growth and effects on the flow field [17–19]. The multiscale nature of thrombosis represents a significant challenge for the development of efficient modelling approaches. A computationally efficient model containing a minimal level of details of thrombotic pathways is needed in order to extend the application of these models to clinically relevant studies.

In this study, we present further refinement and application of an integrated computational model that is capable of predicting thrombus formation in aortic dissections under physiologically realistic flow [20]. The model predictions are based on the distribution of time-averaged haemodynamic parameters and a small number of key transport equations, in order to reduce the computational demand while incorporating thrombus growth and realistic boundary conditions. The model, previously tested in idealized two-dimensional dissection geometries and a three-dimensional backward-facing step [20], has been applied to a patient-specific type B aortic dissection reconstructed from medical images, and predicted patterns of thrombus formation and growth are compared with multiple follow-up CT scans of the same patient. Our results show good agreement between model predictions and *in vivo* observations, demonstrating the applicability of our approach to clinical studies.

## 2. Methods

### 2.1. Thrombosis model

**Figure 1.**
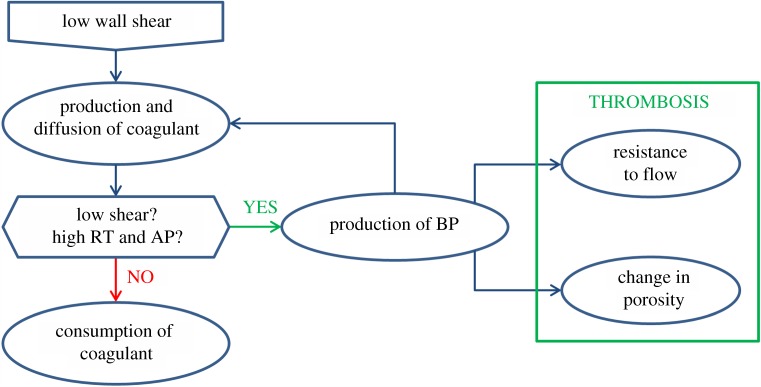
Feedback loop regulating thrombus formation and its effects on blood flow.

Thrombus formation was simulated by using the haemodynamics-based model presented by Menichini & Xu [20]. The computational model is based on transport equations for fluid residence time (RT), resting and activated platelets (RP and AP), and a coagulant (C), whereas a growing thrombus is identified through the local concentration of bound platelets (BP). The transport equations have been simplified in specific regions for more efficient computation, and thrombus growth is controlled through a feedback loop involving C and BP, as outlined in figure 1. Details of the mathematical equations can be found in [20], but a number of important changes have been made which are described below.

For the transport of coagulant, a fixed coagulant concentration was applied at the walls in our previous model. This has been replaced with a flux boundary condition, with the flux depending on the local time-averaged wall shear stress (TAWSS) of the previous cardiac cycle and the local concentration of BP:2.1
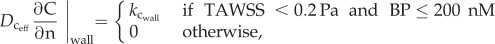
2.2
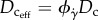
2.3
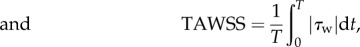
where 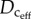 is the effective coagulant diffusivity [20], *T* is the cycle period and 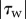 is the magnitude of instantaneous local wall shear stress. The 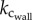 value is fixed at 20 nmol m^−1^ l^−1^ s^−1^. The flux becomes zero when complete thrombosis is achieved (BP > 200 nM). Thrombus growth on the walls can only occur if local TAWSS is lower than 0.2 Pa, and a bulk shear rate threshold 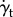 of 50 s^−1^ is adopted for thrombus growth away from the wall. In addition, a consumption term is introduced in the coagulant transport equation, in order to prevent accumulation of *C* in regions of high shear2.4
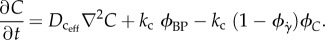


Definitions of symbols and parameter values are given in table 1.

**Table 1.** Definition of symbols and parameter values.

symbol	parameter	value	units	source
*ɛ*	clot porosity		—	
*k*_c_	coagulant kinetic constant	200	nmol l^−1^ s^−1^	[20]
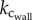	coagulant wall-kinetic constant	20	nmol m^−1^ l^−1^ s^−1^	
*k*_M_	momentum source constant	10^7^	kg m^−3^ s^−1^	[20]
*D*_c_	coagulant diffusivity	10^−8^	m^2^ s^−1^	[20]
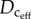	effective coagulant diffusivity		m^2^ s^−1^	
BP_t_	BP threshold	20	nmol l^−1^	[20]
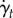	shear rate threshold	50	s^−1^	
*ϕ*_BP_	switching coefficient for BP		—	[20]
*ϕ*_c_	switching coefficient for coagulant		—	[20]
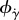	switching coefficient for shear rate		—	[20]
*S*_M_	momentum source		kg m^−2^ s^−2^	

Also included in the model is transport within formed clots which are treated as porous media, with their porosities *ɛ* varying between 0.75 for completely formed clots and 1 for no-thrombus [21], depending on the BP concentration2.5
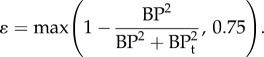
where BP_t_ is a threshold value fixed at 20 nmol l^−1^. In addition, the momentum equation has been modified to account for the effects of time-varying porosity and includes a momentum source proportional to the concentration of BP to account for the effects of thrombus growth on the flow field2.6
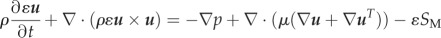
and2.7
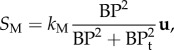
where the vector **u** is velocity, *t* is time, *ρ* is blood density, *p* is pressure and *μ* is blood viscosity. The momentum source *S*_M_ accounts for the resistance imposed by clots on blood flow. This modification replaces the hyperviscous model adopted in our previous work [20] to simulate the solid nature of clots.

### 2.2. Model geometry

**Figure 2.**
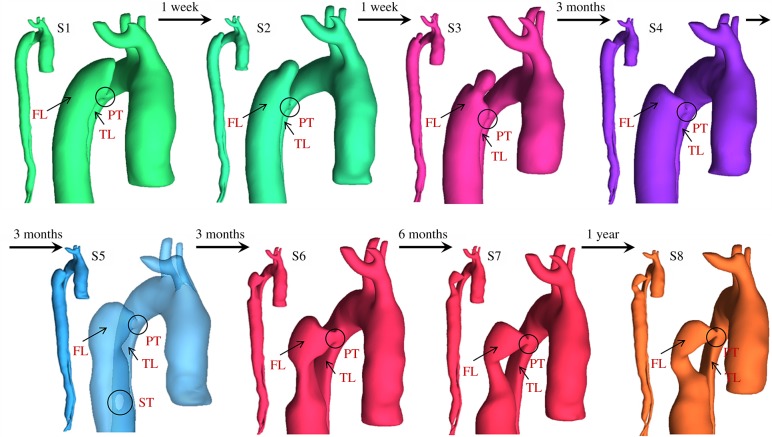
Geometrical descriptions of the aortic dissection at eight follow-up stages. The primary entry tear (PT), true lumen (TL), false lumen (FL) and second tear (ST) are indicated.

A 44-year-old male patient presenting with acute uncomplicated aortic dissection extended beyond the iliac bifurcation was analysed in this study. The patient was given medical treatments and kept under surveillance after being released from the hospital. Follow-up CT scans at eight different stages during an overall period of 3 years showed spontaneous formation of thrombus in several regions of the FL (figure 2). Two different phases could be clearly distinguished: in early stages, thrombus formation was initiated at the top of the FL and expanded in the proximal region (figure 2; S1–S4); in a second phase, lateral thrombosis of the FL was observed, which appeared to be associated with the formation of a second tear in the middle region (figure 2; S5–S8).

**Figure 3.**
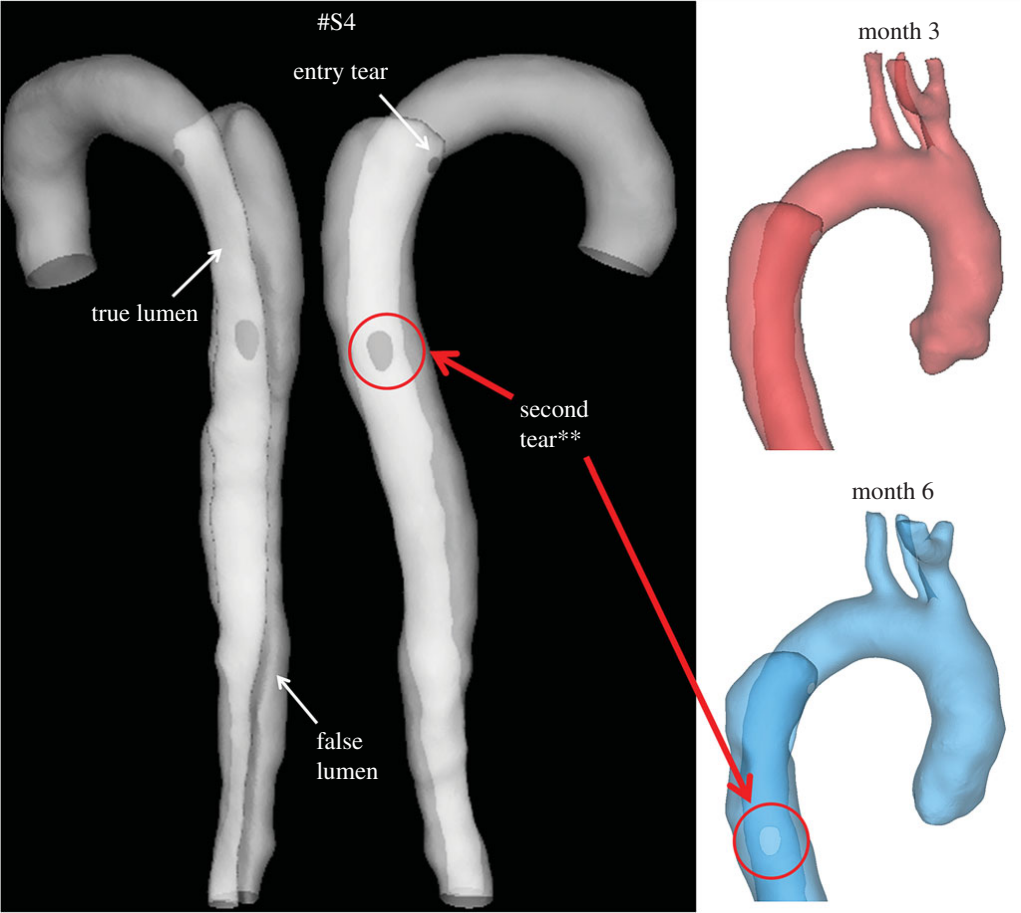
Transparent view of the model S4, showing true lumen, false lumen, and tears. (Asterisks) The original model S4 (top right, ‘month 3’) was modified by artificially introducing a second tear based on the follow-up model S5 (bottom right, ‘month 6’).

Two computational models of the dissected aortas were reconstructed from CT scans by using the image processing software Mimics (Materialise HQ, Leuven, Belgium): one corresponding to the first set of CT scans collected on admission (S1), and one corresponding to the end of the first phase (S4) in order to test the model's capability to predict the lateral thrombus observed in the second phase (S5–S8). Although images acquired at S4 did not show the presence of a middle tear, it was clearly visible at S5, suggesting that the middle tear was formed during the period between S4 and S5, but no scan was performed to capture the exact moment. To overcome this problem, a middle tear was artificially introduced in the second geometry (figure 3) based on information extracted from S5. As the primary aim of this study was to predict thrombus formation within the FL, all the aortic branches were excluded, and those regions were assumed to be non-thrombotic.

The two geometries were meshed using ICEM 15 (Ansys Inc.) and discretized into unstructured grids of approximately 3 million elements each comprising a tetrahedral core with a maximum element size of 1.5 mm and 10 prismatic layers near the walls. The prismatic layers were necessary to control mesh resolution in the boundary layer and ensure convergence of the near-wall transport equations. Mesh sensitivity tests were performed together with local mesh refinement around the tears to obtain a final mesh that was deemed sufficient for mesh-independent solutions.

### 2.3. Computational details

The patient-specific models were implemented in Ansys CFX 15 and simulated for a total of 20 cardiac cycles. Blood was treated as a non-Newtonian fluid described by the Quemada viscosity model [22], and the flow was assumed to be laminar. The model variables were initialized at steady state with a physiological concentration of 2.5 × 10^8^ platelets ml^−1^, a background activation level equivalent to 5% of the inlet platelet concentration [10] (further details and justification are given in §2.4) and zero concentration for all other species.

A realistic flow waveform was applied at the inlet with a flat profile and a period of 1.054 s and a Womersley number of 20.8 [5]. Zero relative pressure was imposed at the outlet [5], which was located slightly above the iliac bifurcation. The walls were assumed to be rigid with no slip conditions. A physiological concentration of 2.5 × 10^8^ platelets ml^−1^ was specified at the inlet, with zero concentration for all other species. The system was simulated for two cardiac cycles to obtain a periodic flow solution and to initialize the model variables, and the thrombus model was introduced from the third cardiac cycle. The results were analysed with the post-processing software CEI Ensight v. 10.

For model S4, an initial wall coagulant concentration equivalent to 1% of the maximum value observed in the patient model S1 was specified to account for the convective effects which are neglected in our thrombus model [20]. Coagulant consumption and production were allowed from the second cardiac cycle, before introducing the actual thrombosis model. This allowed the coagulant concentration to build up in regions of high viscosity and high residence time, or to drop back to zero. As the top region of the FL in S4 was already thrombosed, the top FL surface was specified as thrombus, with a fixed coagulant flux.

### 2.4. Mechanical activation of platelets

A background activation level equivalent to 5% of the inlet platelet concentration was assumed [10,16] on the basis of a potentially thrombogenic surface caused by the tearing of the aortic wall and mechanical activation of platelets by the high shear stress experienced in proximity of the tears. This assumption was evaluated by using the Lagrangian-based model of shear-induced platelet activation introduced by Grigioni *et al*. [23] and calibrated through *in vivo* experiments by Nobili *et al*. [24]. The model quantifies the cumulative load history acting on platelets exposed to time-varying shear stress levels. This is represented by the platelet activation state (PAS) index, which (for the *k*th trajectory) can be calculated as2.8
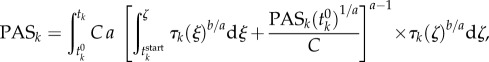
where *t_k_* represents the exposure time, *τ_k_* is the time-varying shear stress load, 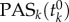 is the initial platelet activation value, which was assumed to be zero. The constants *a*, *b* and *C* were taken from Nobili *et al*. [24] with *a* = 1.3198, *b* = 0.6256 and *C* = 10^−5^. This function was integrated over the trajectories of particles emitted from the inlet at four different time points in a cardiac cycle: beginning of systole, mid-systolic acceleration, peak systole and mid-systolic deceleration. The particle trajectories were created in Ensight and imported into a user-developed Python code in order to integrate the PAS function over the particle pathlines. The results were then visualized in CEI Ensight 10.

## 3. Results

### 3.1. Platelet activation

**Figure 4.**
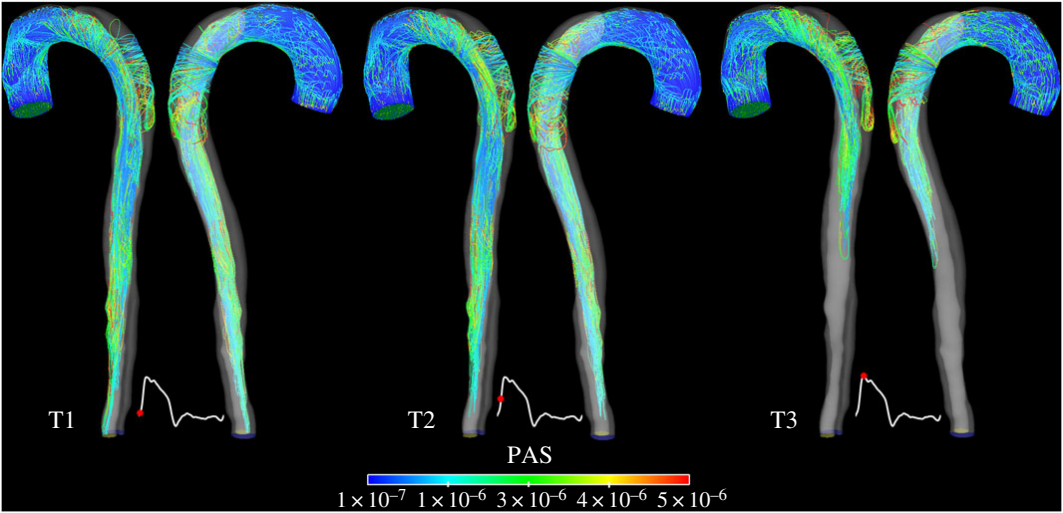
Pathlines of particles ejected at three different time points over the cardiac cycles in the model S1. The trajectories are colour-coded with local PAS values and show the effects of mechanical shear load history on platelet activation.

Figure 4 shows colour-coded platelet-like particles emitted from the inlet at three different time points over a cardiac cycle and tracked for 10 cycles. Activation levels compare well with values previously reported in the literature [25]. As expected, the highest activation levels are observed for those platelets crossing the entry tear and transported into the FL. Platelets emitted during early to peak systole are more likely to be mechanically activated, as there is more flow diverted through the tear in systole than in diastole. The high residence time experienced in the FL and the presence of flow recirculation and stasis will then provide an ideal environment for deposition of activated platelets. All these observations support our assumption that the action of high shear stress in the tear region may have stimulated the initial platelet activity, justifying an initial non-zero concentration of activated platelets.

### 3.2. Wall shear and residence time distribution

**Figure 5.**
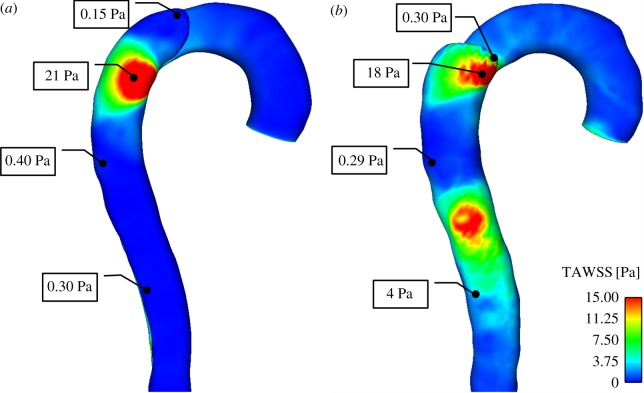
TAWSS distribution in (*a*) S1, and (*b*) S4, before thrombus growth is initiated.

Large spatial variations in TAWSS can be observed in both geometries (figure 5). High TAWSS occurs in the proximity of the tears, where flow is diverted into the FL at high velocity. As discussed in §3.1, these high shear values could possibly stimulate cell activity and promote platelet activation. Much lower shear stress values are found in the top FL region in the model S1, corresponding to a high residence time and viscosity. In the model S4, shear stress below 0.4 Pa is observed in the top FL and in-between the two tears. The formation of a second entry tear clearly affects the flow pattern in the region between the tears, resulting in reduced wall shear stress. As thrombus grows in the top FL, the shear stress in the middle region is reduced even further, leading to the formation of a new thrombus.

**Figure 6.**
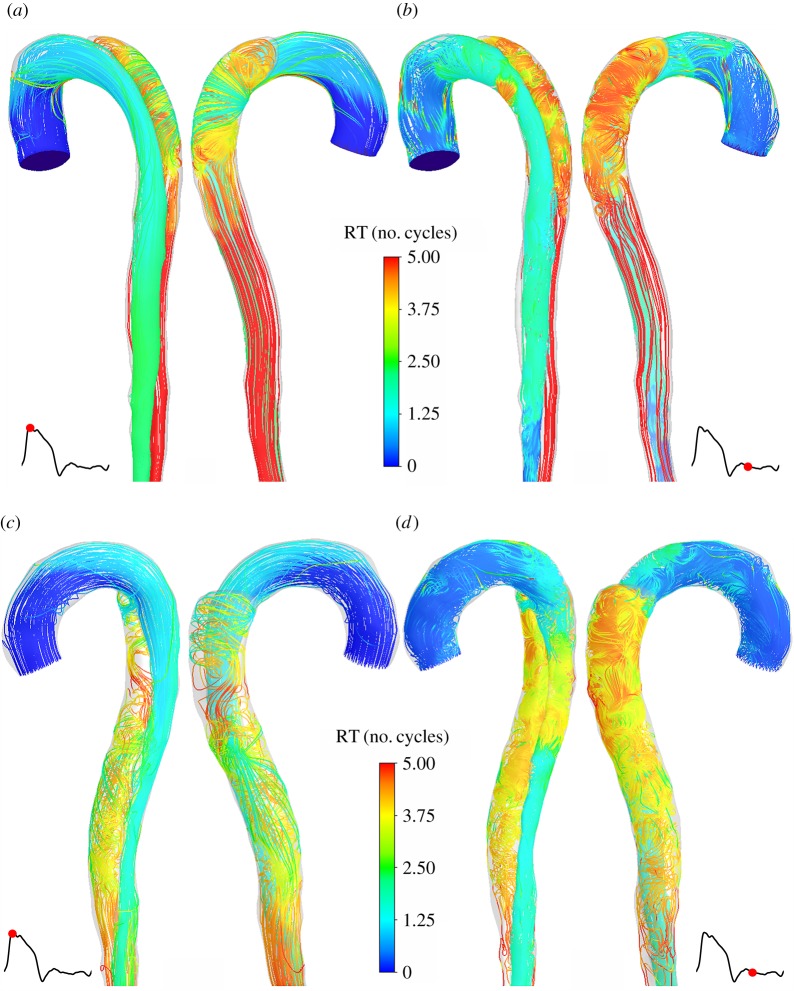
Instantaneous streamlines at peak systole in S1 (*a*) and S4 (*c*), and in mid-diastole in S1 (*b*) and S4 (*d*), showing the local RT distribution, expressed in number of cardiac cycles.

Distributions of normalized RT, expressed in number of cycles, at peak systole and mid-diastole in S1 and S4 are presented in figure 6. In both models, fluid residence time above five cardiac cycles is observed in parts of the FL, suggesting partial flow stagnation there. The formation of a second tear alters flow features in the FL resulting in more chaotic flow patterns and a reduction in local fluid residence time when compared with model S1. RTs in the true lumen (TL) are within the normal range when compared with a baseline value defined as *L*/*v*_mean_, representing the characteristic timescale of blood convection through the system, where *L* is the distance travelled along the centreline, and *v*_mean_ is the mean cycle velocity. During the diastolic phase, fluid particles with high RTs are transported from the FL into the TL. In the model S4, a recirculation zone between TL and FL is also created between the two tears (figure 6*d*). These recirculating fluid particles characterized by a high RT are then transported away from the domain in systole (figure 6*c*).

### 3.3. Thrombus growth over time

**Figure 7.**
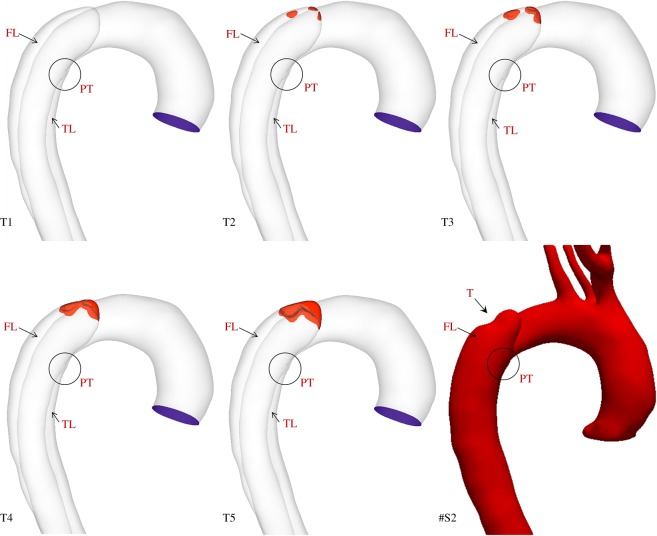
Thrombus formation over time at T1 = 2 s, T2 = 6 s, T3 = 10 s, T4 = 15 s, T5 = 20 s, compared with subsequent thrombus formation observed in follow-up geometry S2 extracted from CT scans acquired after one week. FL, false lumen; TL, true lumen; PT, proximal tear; T, thrombosed region.

**Figure 8.**
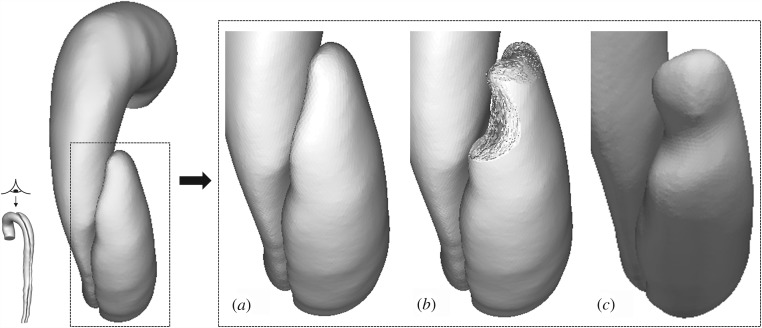
Comparison between predicted lumen surface in S1 and follow-up geometry S2. (*a*) Original lumen surface in S1, (*b*) predicted lumen surface after thrombus growth, (*c*) lumen surface in follow-up geometry S2, extracted from images acquired one week after S1.

Thrombus formation is initiated in regions of low shear stress, high residence time and high concentration of AP. As expected, the top FL region in the model S1 is gradually thrombosed over time (figure 7). Thrombosis is initiated at different locations, and expands towards the proximal tear, showing good qualitative agreement with *in vivo* observations (figure 7 S2 and figure 8).

**Figure 9.**
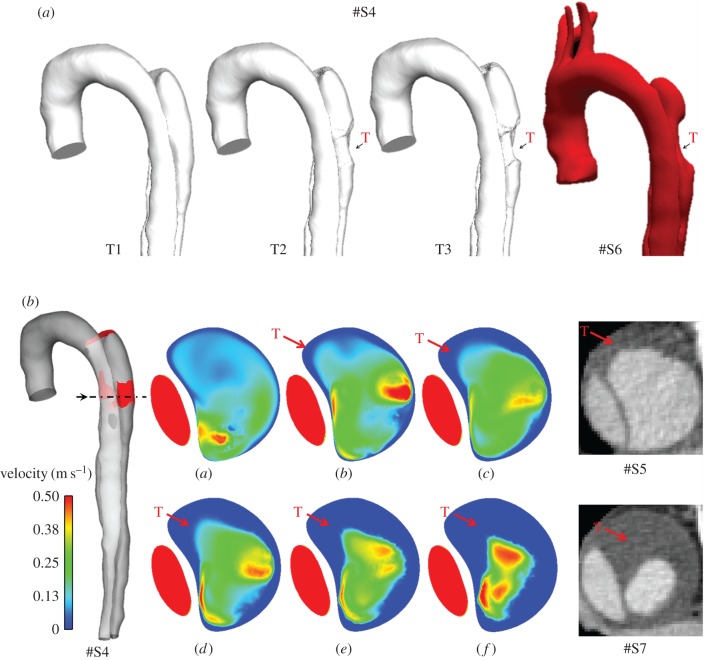
(*a*) Time evolution of the fluid domain at T1 = 3 s, T2 = 14 s, and T3 = 21 s, compared with subsequent thrombus formation (T) observed in follow-up geometry S6, extracted from CT scans acquired six months after S4. (*b*) Velocity magnitude contours at peak systole just above second tear showing the effects of thrombus growth (T) on the flow field at different time points (*a*) *t* = 3.25 s, (*b*) *t* = 7.47 s, (*c*) *t* = 9.58 s, (*d*) *t* = 13.8 s, (*e*) *t* = 15.9 s, and (*f*) *t* = 21.2 s, compared with follow-up CT scans from models S5 and S7, taken, respectively, after three and nine months from S4. The velocity in the thrombosed region is gradually reduced as thrombus is formed. In the model S4 (left), in red, thrombus.

In the model S4, the presence of a second tear affects flow patterns in the region in-between the two tears, and causes a local fall in wall shear stress on the lateral walls, resulting in a gradual formation of thrombi in multiple locations. As shown in figure 9*a*,*b*, there is a good qualitative agreement between model predictions and follow-up CT scans of the patient. In particular, the predicted location and shape of thrombus on the lateral FL side agree very well with the geometry reconstructed from images acquired at S6 (figure 9*a*), although comparisons at a transverse plane (figure 9*b*) reveal some differences near the flap (the thin wall separating the true and FL).

## 4. Discussion

The role of haemodynamics in thrombus formation has been investigated by many researchers. Studies by Sheriff *et al*. [26] and Nesbitt *et al*. [27] emphasized the importance of shear gradients in the development of thrombosis, showing that thrombus formation is generally associated with rapid changes in blood flow and preferentially takes place in regions of low shear [15]. Nesbitt *et al*. [27] also revealed that platelet aggregation is primarily driven by changes in rheological properties of blood and local haemodynamics, with soluble agonists having a negligible role. Based on these findings, we have developed a haemodynamics-based predictive model for thrombosis in patient-specific dissections under pulsatile flow. This is achieved by using haemodynamics-related parameters as surrogates so as to bypass the need for simulating the complex chain of biochemical processes. The model simulates the coagulation process by using a coagulant that forms on the walls exposed to high residence time and low shear. The transport of coagulant simulates the expansion of the reaction zone, which is characterized by low Peclet numbers and diffusion-limited reactions. To incorporate the effects of convection on thrombus growth, saturation functions controlled by time-averaged shear rates are introduced to turn the process on and off depending on the local haemodynamic environment. The model has been previously tested on idealized two-dimensional aortic dissection models and a three-dimensional backward-facing step [20], demonstrating good agreement with expected trends and experimental observations from Taylor *et al.* [28]. Here, we have demonstrated its applicability to realistic geometries and pulsatile flow.

### 4.1. Platelet activation

In order to evaluate the likelihood for shear-induced activation, the PAS function accounting for the mechanical loading history acting on blood cells is integrated over particle trajectories in S1. Our results show that particles passing through the tear are subject to a high shear load, which can potentially stimulate cell activity and promote subsequent adhesion on the walls. As flow in the FL is partially stagnant, it is likely that those particles previously exposed to high levels of shear will be trapped in flow recirculation regions, leading to thrombosis in regions of high viscosity and low shear stress. The initial inlet concentration of AP was fixed at 5% of the inlet concentration of RP. This value, previously proposed by Sorensen *et al*. [11] and also adopted by Anand *et al*. [29], Bedekar *et al*. [30] and Taylor *et al*. [16], allows us to reach local AP concentrations needed for the formation of BP within a short time frame, while still preventing high AP concentrations in non-thrombotic regions. As BP is the only variable directly dependent on local AP values, results are not particularly sensitive to the initial AP concentration. Changes in local BP concentrations are linearly proportional to variations in AP levels [20], thus only large changes in the initial AP concentration by several orders of magnitude would significantly affect BP values and hence thrombus formation and growth.

### 4.2. Identifying regions of thrombus formation

Analysis of flow at different follow-up stages has shown an association between thrombus formation and low TAWSS, which is used as a key parameter to initiate thrombosis on the wall. Other low-shear based models have been reported by Harrison *et al*. [31], Zimny *et al*. [32] and Malaspinas *et al*. [14]. Regions of particularly low shear stress are associated with high residence time and increased blood viscosity, which favour cell–cell interactions and increase the likelihood for cell adhesion on vessel walls [33]. Because the predicted thrombus formation is sensitive to the chosen wall shear threshold, it is important to identify a suitable value for clinical applications. From the patient-specific analysis of haemodynamic parameters at different follow-up stages and subsequent CT scans, a single TAWSS threshold of 0.2 Pa was found to be capable of predicting the exact locations of thrombus formation. A lower threshold would cause slower growth, whereas higher thresholds were found to over-predict thrombus growth, especially for threshold values greater than 0.25 Pa. However, no significant differences were found in the location of thrombus formation and its growth pattern. Nevertheless, further studies on a large cohort of patients will be required in order to test the validity of this threshold value for general applications. On the other hand, experimental evidence demonstrates that particularly high shear stress might also play an important role in thrombosis by stimulating local platelet aggregation via von Willebrand factor, and several computational models have been proposed to simulate this process [34,35]. Our results appear to indicate that the low-shear thrombotic pathway is more important in FL thrombosis in type B aortic dissection. Nevertheless, it would be desirable to also examine the role of high-shear thrombotic pathways for a more complete description of thrombus formation.

### 4.3. Thrombus growth

With artificially accelerated kinetics [20], the process has been simulated over 20 cardiac cycles, which is much shorter compared with the real timescale for thrombosis in dissection. This acceleration is possible as regions of thrombus growth are generally associated with relatively low velocities, so that growth only causes gradual changes in flow. Similar approaches have been adopted by others [13,14,36]. By neglecting convection and considering the time-averaged effects of haemodynamic parameters such as shear rates and residence time, regions of potential thrombosis can be identified, whereas interactions between blood flow and thrombus growth can also be accounted for. The effects of thrombus growth on the flow field are captured through a momentum source term and changes in local porosity [12,13]. While most of previous models assumed steady flow [10,12,16,37], we found that areas of flow recirculation and low shear could be better captured through time-averaged variables under pulsatile flow. The use of time-averaged values can also reduce the dependence of the process on inlet boundary conditions that are subject to large variations over the actual time course of thrombus growth.

Comparisons with follow-up scans taken from the patient at different time points have demonstrated that our model is able to capture the formation of thrombus and its expansion in the proximal region of FL during the initial phase (model S1); thrombosis started on the outer wall and gradually expanded towards the entry tear. The growth of thrombus on the sidewalls of FL during the second phase was also reasonably well predicted by including a newly formed middle tear in model S4, which altered flow features and caused a drastic reduction in shear stress and the formation of a flow recirculation region proximal to the new tear. Also, as the CT scans for model S4 were acquired more than three months after the patient showed the first signs of thrombosis, it is reasonable to assume that, if other potentially thrombotic regions existed, coagulation factors produced within the forming thrombus and transported by the flow could have slowly built-up near the walls. This reasoning justified an initial non-zero concentration of coagulant on the walls, which was applied two cardiac cycles before running the thrombus model, in order to allow wall concentrations to drop back to zero in non-thrombotic regions. Simulated thrombus growth patterns are in good agreement with follow-up data. Some discrepancies were observed near the flap side, where the predicted distance between the thrombus and the proximal tear was larger than that measured from subsequent CT scans.

Overall, the results obtained from our simulations show satisfying agreement with patient-specific follow-ups, although this study has several limitations. The use of time-averaged variables, the rigid wall assumption and the exclusion of aortic branches may affect the distribution of haemodynamic parameters [38,39], thereby influencing the overall prediction of thrombus growth. In particular, the intimal flap between the TL and FL may be subject to motion driven by the pressure difference between the two chambers. The effects of wall compliance and viscoelastic behaviour of blood clots should be investigated in future studies. Other limitations involve the assumptions made in creating the model geometry. Model S4 was virtually modified by adding a second tear extracted from follow-up scans acquired three months later. During the interval between S4 and S5, the patient geometry experienced significant changes, including enlargement of the tear diameter, thrombus growth in the proximal region and formation of microtears on the flap. Although these changes are likely to affect local flow patterns and shear distribution, a comprehensive wall model would be required to solve simultaneously the deformation of the TL and FL, and to predict any potential expansion or rupture, which is beyond the scope of this study.

Lastly, this study focuses on predicting the possibility and extent of FL thrombosis rather than the duration of the process. Thrombus growth is accelerated in order to improve computational efficiency, so that simulation of FL thrombosis can be achieved in a feasible time frame (e.g. 20 cardiac cycles in this study). A similar strategy has been adopted by others to simulate thrombosis in cerebral aneurysm [13,14]. The relationship between our simulated timescale and real timescale was examined, using a backward-facing step under steady flow. The simulation results obtained with an augmentation factor of 150 for coagulant diffusivity were compared with experimental data [28], and there was a good agreement between predictions obtained after a simulation time of 40 s and measurements taken at 90 min, suggesting that the simulation time could be multiplied by the augmentation factor (40 × 150 = 6000 s) to roughly estimate the equivalent real time (90 min = 5400 s). However, such a simple relation may not apply to pulsatile flow, as the interface between diffusion-dominated and convection-dominated domains hinders the transport of proteins and coagulation factors to the clot surface. Given that a number of patient-related and environmental factors might also affect thrombus growth rates, it is not possible to deduce what relationship exists between our simulated timescale and real-time thrombus growth from a single patient case study. Multiple patient-specific case studies and comparisons with experimentally derived biochemical models are required to elucidate the relationship between the two different timescales.

## 5. Conclusion

This study represents the first attempt to predict FL thrombosis in patient-specific type B aortic dissections. A computationally efficient haemodynamics-based model is applied to a patient-specific case and validated against *in vivo* data collected at eight follow-up scans over 3 years. The two separate stages in thrombus growth observed from CT scans are well captured by the computational model, which is able to predict initial growth of thrombus in the proximal region and subsequent lateral thrombosis caused by the formation of a second tear. Future studies will include applying this computational model to more medically and endovascularly treated dissection patients in order to further validate and refine the model. Once fully validated, the model can be used to predict FL thrombosis based on patient-specific information at the time of initial diagnosis, which would provide great assistance in clinical decision-making.

## Data accessibility

Data supporting this publication can be obtained on request from biofluids-and-transport@imperial.ac.uk.
